# Progress of Epidemics in Forty-One Towns and Districts

**Published:** 1857-10

**Authors:** 


### PROGRESS OF EPIDEMICS.

### LOCAL REPORTS OF EPIDEMIC AND ENDEMIC DISEASES

*During the Months of June, July, and August,* 1857.

| Place. | County. | Lat. | Long. | Observer. |
|---|---|---|---|---|
| St.Mary, Scilly | Cornwall - | 49.50 N. | 6.24 W. | J. G. Moyle, Esq. |
| Teignmouth - | Devonshire - | 50.32 N. | 3.29 W. | W. C. Lake, Esq. |
| Odiham - - | Hampshire - | 51. 8 N. | 1. 3 W. | J. McIntyre, M.D. |
| Canterbury - | Kent - - | 51.17 N. | 1. 4 E. | G. Rigden, Esq.<br>W. Haffenden, Esq. |
| Chatham - | Kent - - | 51.21 N. | 0.14 E. | F. J. Brown, M.D. |
| Wandsworth - | Surrey - - | 51.28 N. | 0. 7 W. | G. E. Nicholas, Esq. |
| Putney - - | Surrey - - | 51.28 N. | 0. 8 W. | R. H. Whiteman, Esq. |
| Up. Holloway | Middlesex - | 51.32 N. | 0.03 E. | W. B. Kesteven, Esq. |
| Wanstead - | Essex - - | 51.32 N. | 0. 2 W. | F. Collins, M.D. |
| Swansea - | Glamorgansh. | 51.38 N. | 3.50 W. | W. H. Michael, Esq. |
| Aspley Guise - | Bedfordshire - | 52. 1 N. | 0.37 W. | J. Williams, M.D. |
| Saffron Walden | Essex - - | 52. 3 N. | 0.12 E. | H. Stear, Esq. |
| Bedford - - | Bedfordshire - | 52. 8 N. | 0.51 W. | T. H. Barker, M.D. |
| Newpt.Pagnell | Buckinghams. | 52.10 N. | 0.42 W. | G. O. Rogers, Esq. |
| Sharnbrook - | Bedfordshire - | 52.12 N. | 0.40 W. | R. S. Stedman, Esq. |
| Wellingbro' - | Northamptons. | 52.20 N. | 0.40 W. | B. Dulley, Esq. |
| Beccles - - | Suffolk - - | 52.25 N. | 1.48 E. | W. E. Crowfoot, Esq. |
| Thetford - | Norfolk - - | 52.26 N. | 0.45 E. | H. W. Bailey, Esq. |
| Stourbridge - | Worcestersh. - | 52.28 N. | 2.32 W. | A. Freer, Esq. |
| Dudley - - | Staffordshire - | 52.30 N. | 2.10 W. | J. H. Houghton, Esq. |
| Barrowden - | Rutland - | 52.34 N. | 0.38 W. | H. J. Swann, Esq. |
| Wisbeach - | Cambridgesh. | 52.39 N. | 0. 5 E. | W. H. Hole, Esq. |
| East Dereham | Norfolk - - | 52.40 N. | 0.57 E. | J. Vincent, M.D. |
| Pontesbury - | Shropshire - | 52.43 N. | 2.50 W. | Wm. Eddowes, Esq. |
| Nottingham - | Nottinghamsh. | 52.50 N. | 1.10 W. | T. Robertson, M.D. |
| Burton-on-Trt. | Staffordshire - | 52.53 N. | 1.53 W. | S. Thomson, M.D. |
| Oswestry - | Shropshire - | 52.53 N. | 2.59 W. | P. Cartwright, Esq. |
| Wrexham - | Denbighshire - | 53. 2 N. | 3. 1 W. | E. Williams, M.D. |
| Hawarden - | Flintshire - | 53.11 N. | 3. 2 W. | T. Moffat, M.D. |
| Lincoln - | Lincolnshire - | 53.12 N. | 0. 5 W. | S. Lowe, Esq. |
| Alford - - | Lincolnshire - | 53.15 N. | 0. 6 E. | R. U. West, M.D. |
| Gainsborough | Lincolnshire - | 53.23 N. | 0.47 W. | D. Mackinder, M.D. |
| Liverpool - | Lancashire - | 53.24 N. | 2.59 W. | Thos. Bickerton, Esq. |
| Warrington - | Lancashire - | 53.25 N. | 2.32 W. | C. N. Spinks, Esq. |
| Wigan - - | Lancashire - | 53.32 N. | 2.33 W. | Jephtha Hussey, Esq. |
| Bolton - - | Lancashire - | 53.35 N. | 2.19 W. | W.H. Pendlebury, Esq. |
| Bramley - | Yorkshire - | 53.49 N. | 1.38 W. | J. N. Radcliffe, Esq. |
| York - - | Yorkshire - | 53.58 N. | 1. 3 W. | W. Procter, Esq. |
| Wst. Auckland | Durham - | 54.45 N. | 1.40 W. | G. Todd, Esq. |
| Rothbury - | Northumberld. | 55.25 N. | 1.50 W. | E. C. Summers, Esq. |
| Lerwick - | Shetland Isles | 60. 9 N. | 1. 8 W. | G. W. Spence, M.D. |

### QUARTERLY STATEMENT—No. XI.

[*The dates denote that the disease appeared in the weeks then ending.*]

SCARLET FEVER.

Odiham—August 21-28
Canterbury—All July and August
Newport Pagnell—June 19, July 10
Beccles—August 21-28
Stourbridge—July 3
Dudley—June 5, 19, July 3, Aug. 7-14
Wisbeach—August 14-28
East Dereham—All August
Pontesbury—July 24-31
Burton-on-Trent—All June and July, [Aug. 7
Hawarden—July 4-11, Aug. 1

Lincoln—July 24-31
Gainsborough—July 17-31, all Aug.
Liverpool—Every week
Warrington—All June, July 3-17, Aug.
Wigan—July 10 [14-28
Bolton-le-Moors—June 5-19, July 10-
Bramley—July 3 [31, Aug. 14-21
York—July 10-24, August 7-21
West Auckland—All July and August

MEASLES.

Odiham—August 14-21
Stourbridge—June 12
Dudley—June 5-12, July 10-24
Barrowden—July 24, August 7
Pontesbury—June 5, all August
Burton-on-Trent—August 28
Wrexham—June 12-26, July 3-10
Lincoln—All June, July 3
Gainsborough—All June, July 3-17
Liverpool—Every week
Warrington—All June & July, Aug. 7-14
Bolton-le-Moors—June 12-19, July 17-31, August 7-14
York—June 12-26, July 3, 17, Aug. 7-14
Lerwick—All June, July 3

SMALL-POX.

Chatham—August 14-28
Swansea—July 17, August 7-14
Thetford—June 5, 19-26, July 3-17
Stourbridge—Every week
Dudley—June 5-12, 26, July 17-31, Aug.
Wisbeach—July 10-31, Aug. 7 [14-28
East Dereham—All June
Pontesbury—All June, July 3-10
Oswestry—All June & July, August 7
Wrexham—June 12-26, July 3-10
Lincoln—July 17-24
Liverpool—Every week
Warrington—July 10-17, 31, August 7
Wigan—June 19, July 17, August 7
Bolton-le-Moors—June 5-19, July 24-31, August 7-14
York—June 12-26, July 10-24, Aug. 7-14

HOOPING-COUGH.

Chatham—July 3-17, August 14-28
Upper Holloway—July 17-31, Aug. 7
Swansea—June 12-19, August 14-28
Saffron Walden—June 19
Bedford—July 10-31, August 7-14
Newport Pagnell—June 5-12, July 24-31, August 7-21
Sharnbrook—Every week
Stourbridge—June 12, 26, July 17-31
Dudley—June 12, July 10, August 21
Barrowden—June 19
Wisbeach—June 5-19, August 21-28
Pontesbury—Every week
Burton-on-Trent—All July & August
Oswestry—Every week
Lincoln—June 5-19
Gainsborough—July 31, August 7
Liverpool—Every week
Warrington—Every week
Wigan—June 5-19, July 3, 24
Bolton-le-Moors—June 19-26, August
Bramley—June 5 [14-28
York—June 19, July 17

CROUP.

Canterbury—June 19
Chatham—July 3
Swansea—July 17
Thetford—June 26, July 10, 31
Stourbridge—July 17
Dudley—July 24
Oswestry—June 5-12, July 17
Lincoln—June 26, July 3-17
Liverpool—June 12, July 17
Warrington—June 19
Wigan—July 17, August 14
Bolton-le-Moors—July 17 24, Aug. 28
Rothbury—July 3

CATARRH.

Teignmouth—June 5-12, 26, July 3-10, 31 [Aug. 14-28
Odiham—June 5-12, July 10-17, 31,
Chatham—June 26, July 3-17 August 7-21
Wandsworth—June 5-12, July 3, 24-31
Putney—June 5-19
Upper Holloway—July 3-24
Swansea—June 19-26, July 17-31, all
Saffron Walden—July 3-10 [Aug.
Sharnbrook—June 19-26, all July, Aug. 7-14, 28
Wellingborough—June 5, 19, all Aug.
Beccles—June 12, 26, July 24-31
Thetford—June 5, 19-26, July 3, 24, August 7, 31
Stourbridge—June 19, July 31 all Aug.
Dudley—June 12, July 14
Wisbeach—July 17-24
Pontesbury—July 24-31
Burton-on-Trent—All August
Gainsborough—July 24-31, all Aug.
Liverpool—Every week
Bolton-le-Moors—June 12-19, July 3-
Bramley—June 12 [24
Rothbury—July 17
Lerwick—June 5-19, July 3-10, Aug. 7

INFLUENZA.

Scilly Islands—June 5-19
Teignmouth—June 5-19
Chatham—July 3, 31
Swansea—June 26, August 14
Saffron Walden—July 24
Newport Pagnell—All June and July, August 21-28
Beccles—June 5, July 3-10
Thetford—June 12-19, July 3-10, 24, August 7-14

Pontesbury—June 5-12, August 21-28
Lincoln—July 24-31, August 7
Liverpool—All June, July 3-10, 31, August 7-14, 28
Bolton-le-Moors—June 26, July 3
York—August 14-21

ERYSIPELAS.

Canterbury—July 10, 24, August 21
Chatham—July 3, 17-24, August 7
Wandsworth—June 5, July 3, 31
Swansea—June 19-26, July 31, Aug. 7
Bedford—August 21-28
Newport Pagnell—July 31, all August
Sharnbrook—August 21
Beccles—July 17, August 7
Thetford—June 5, August 21
Dudley—June 19, July 3-10, Aug. 21
Barrowden—July 31
Wisbeach—June 12-26
Pontesbury—July 3-10, all August
Burton-on-Trent—June 19, July 31
Oswestry—All August
Alford—June 26, July 31
Gainsborough—All July and August
Liverpool—Every week
Warrington—July 17-24
Bolton-le-Moors—June 19-26, July
Bramley—June 5-12 [17-24
York—June 12, July 31
West Auckland—July 31, all August

CHOLERA (ENGLISH).

Odiham—All August
Chatham—July 31, August 7
Putney—August 14-21
Swansea—August 21-28
Newport Pagnell—August 7-14
Wellingborough—August 14-28
Thetford—August 21-28
Stourbridge—August 28
Barrowden—July 24, August 21
Pontesbury—July 31, August 7-21
Liverpool—June 5, 19 26, July 3, 17, 31, August 14-28
Bolton-le-Moors—Every week
Lerwick—July 17

AGUE.

Canterbury—All June & July, Aug. 28
Chatham—June 5,19-26 all July&Aug.
Putney—July 10
Wanstead—June 26
Aspley Guise—June 5-12
Bedford—June 5-26, July 3, 10
Thetford—June 5, 19, July 10, 31
Dudley—June 5
Barrowden—June 19
Wisbeach—Every week
East Dereham—All July, August 7
Lincoln—July 31, August 7-14
Alford—June 19, July 17
Liverpool—All August

REMITTENT FEVER.

Teignmouth—June 19-26, July 10, 24-
Odiham—July 3 [31, Aug. 14-28
Canterbury—June 12, 26, July 17, 31,
Chatham—June 5, July 10 [Aug.14-28
Putney—June 5, July 10
Newport Pagnell—July 3-24 [7-21
Beccles—June 19-26, July 3-10, Aug.
Thetford—June 19-26
Wisbeach—All June and July, Aug. 7
Burton-on-Trent—July 17, Aug. 28
Oswestry—All July and August
Lincoln—July 10-31
Alford—July 17, August 14-28
Liverpool—All August [28
Wigan—June 12-26, July 17-24, Aug. 7,
Bolton-le-Moors—July 10-24, Aug. 7-14

DIARRHŒA.

St. Mary's, Scilly—July 17-31, Aug. 7-14
Teignmouth—June 12-26, July 3-10, 24-31, August 7-14, 28
Odiham—June 26, all July and Aug.
Canterbury—Every wk. except Aug. 28
Chatham—June 19-26, all July & Aug.
Wandsworth—Every week
Putney—June 5, 26, all July and Aug.
Upper Holloway—July 10-31, all Aug,
Wanstead—June 26, all July and Aug.
Aspley Guise—July 17, 31, all August
Swansea—June 12-26, all July & Aug.
Saffron Walden—June 12, all July & Aug.
Bedford—June 26, all July & August
Newport Pagnell—All July & August
Sharnbrook—July 24-31, all August
Wellingborough—July 17-31, all Aug.
Beccles—June 5-12, all July & Aug.
Thetford—June 26, July 10, 24-31, all August [Aug.
Stourbridge—June 19-26, July 17-31, all
Dudley—June 5-19, all July and Aug.
Barrowden—June 26, July 10-31, all August
Wisbeach—June 5, 26, all July & Aug.
East Dercham—Every week
Pontesbury—June 12-26, all July & Aug.
Burton-on-Trent—Every week
Oswestry—All August
Wrexham—July 24-31, all August
Hawarden—June 5-12, July 3, 17-31, August 7, 21-28
Lincoln—July 24-31, August 7-21
Alford—June 12-19, all August
Gainsborough—July 31, all August
Liverpool—All August
Warrington—All July and August
Wigan—June 19-26, July 3, 17-31, Aug.
Bolton-le-Moors—Every week [7, 21
Bramley—June 19-26, July 17, Aug. 21-
York—Every week except last [28
West Auckland—All July and August

Rothbury—All July, August 7
Lerwick—July 17-24, August 7, 28

DYSENTERY.

St. Mary's, Scilly—August 21
Odiham—July 3, 31, August 14
Canterbury—July 24, August 14
Chatham—July 17, 31, August 14-21
Wandsworth—June 5-19, July 10-17, [Aug, 21-28
Putney—June 5
Upper Holloway—July 31, August 7
Swansea—July 24, August 14-28
Bedford—July 17-31
Newport Pagnell—July 10-17, all Aug.
Wellingborough—August 7, 21
Stourbridge—August 7-14
Wrexham—July 24-31, August 7
Lincoln—July 31, August 7-21
Alford—August 14-28
Gainsborough—August 14-28
Liverpool—Every week
Bolton-le-Moors—June 5-12, July 3-17, 31, all August
Bramley—June 12, 26, July 17, Aug. 14
York—July 3, 17-24, August 7-21

TYPHUS.

Teignmouth—June 26, July 10, 24, August 7
Canterbury—Every week except last
Chatham—June 5, 19-26, all July, August 7-14, 28
Wandsworth—June 19-26, July 3, 17-24, [Aug. 7-14
Putney—June 5, 26
Saffron Walden—July 3
Bedford—August 14 28
Newport Pagnell—July 31, Aug. 7-14
Sharnbrook—August 7
Wellingborough—Every week
Beccles—July 10-31, August 21-28
Thetford—June 19, July 3-10, 24, Aug. 21-28
Dudley—All June, July 3-24, all Aug.
Wisbeach—July 17-31, all Aug.
East Dereham—August 21-28
Wrexham—June 12-26, August 21-28
Lincoln—July 17-31, August 7-21
Alford—August 21
Gainsborough—June 19-26, all July, [Aug. 7-21
Liverpool—Every week
Bolton-le-Moors—June 19-26, Aug. 14-[21
Lerwick—June 12

PUERPERAL FEVER.

Chatham—July 24, August 14
Bolton-le-Moors—June 19-26, July 3-[24
York—August 21

CARBUNCLE.

Canterbury—June 5
Swansea—All August
Saffron Walden—July 3, 24
Bedford—August 14
Newport Pagnell—July 10-17, Aug. 7-[14
Wellingborough—Aug. 21-28
Beccles—June 19
Thetford—July 10
Stourbridge—June 19
Wisbeach—July 3-24
East Dereham—August 21-28
Oswestry—August 14-28
Wrexham—July 10-31
Alford—August 14
Gainsborough—July 10-31
Liverpool—June 5, July 17-24
Bolton-le-Moors—July 17-24
Rothbury—July 3

CHICKEN POX.

Wandsworth—July 17, August 21
Lerwick—June 19, July 31

MUMPS.

Lerwick—June 12

WHITLOW.

Lerwick—June 12-10

TYPHOID FEVER.

Stourbridge—June 12, July 3-10, Aug. [28
Wigan—July 17-24

RHEUMATISM.

Hawarden—June 26

### ADDITIONAL OBSERVATIONS.

*St. Mary's, Scilly.*—Mr. Moyle writes: "We have had a very healthy quarter: a few cases of influenza occurred in June, of diarrhœa in July and August, and one case of dysentery. Ozone has been daily indicated. It has never reached beyond 9 of Schonbein's scale; nor has it been less than 4. The temperature has been remarkably high for these islands, and the weather generally exceedingly fine."

*Teignmouth.* A remarkable feature in the meteorology of the quarter was the small amount of rain and the high temperature, from about June 18 till the end of the quarter. The temperature for the three months was as follows:

| | *June.* | *July.* | *August.* |
|---|---|---|---|
| | Deg. | Deg. | Deg. |
| Highest temperature ............ | 75·4 | 80·0 | 81·0 |
| Lowest temperature ............ | 44·0 | 51·1 | 47·5 |
| Mean of all highests ............ | 68·7 | 71·9 | 72·9 |
| Mean of all lowests.............. | 52·8 | 55·7 | 57·2 |
| Total amount of rain in inches.... | 1·152 | 1·119 | 0·225 |

The quarter was decidedly healthy on the whole. Cases of diarrhœa were few in number, increasing more towards the close of August, but then not to any great extent.

*Odiham.*—Dr. M'Intyre says that the scarlet fever was *imported* from London. The choleraic condition was in some cases severe, but in none fatal. Diarrhœa was general, and was markedly prevalent during increased atmospheric temperature.

*Canterbury.*—Mr. Rigden writes : "Diarrhœa was very prevalent, particularly during the last six weeks, in this city and neighbourhood ; but although seven deaths, in a population of nearly nineteen thousand inhabitants, have resulted from this disease, it has not assumed a malignant type. Four of the deaths were in young children under eighteen months ; the three others were aged, respectively, 84, 63, and 25. The latter is registered simply as 'diarrhœa, 1 day', and occurred in a locality of medium sanitary conditions. Two deaths, during the quarter, have been caused by diphtheritis in children, aged respectively 9 years and 4 years, members of the same family, who resided in a very close and unhealthy locality. Seven deaths, from fever, in persons aged respectively 51, 18, 9, 79, 5, 7, and 8. Two deaths from scarlatina occurred in children aged respectively 7 and 3 years ; one, from thrush, in a child aged 6 weeks ; and one from purpura hæmorrhagica, in a child aged 17 months. Diphtheritis was very prevalent, and malignant, in a village about nine miles from this city. It has been difficult to account for it, unless upon the supposition of defective drainage, or the consumption of water impregnated with deleterious matters."

Mr. Haffenden furnishes the subjoined returns, drawn up by eight observers of the Epidemiological Committee of the East Kent and Canterbury Medical Society :

*Abstract of the returns of Zymotic diseases during the Summer Quarter*, 1857.

"The number of cases returned during the last three months amounted to two hundred and fifty-five, about one half of which come under the head of diarrhœa ; and by far the greater number of these occurred during the last month. Six deaths were registered, during the quarter, from zymotic diseases ; viz., two from diarrhœa, three from fever, and one from typhus. Of scarlatina, twenty-three cases were returned, all of which recovered. It is reported as being mild in character. A few cases of suppurating glands in the neck were mentioned in conjunction with scarlatina; and, in a few instances, anasarca. Measles and chicken-pox were observed, in a few instances, in the months of June and July but quite disappeared during the last month. Diarrhœa was very prevalent during August; but was not much noticed in the early part of the summer. One hundred and thirty-three cases have been returned, independently of a great number that were treated, at the hospital, in a casual way. In two instances it proved fatal,—one from exhaustion; and in the other, a child 13 months of age, the disease was said to put on the choleraic form. Of fever, fifty-six cases were reported: a few of them were remittent, the rest continued. Three terminated fatally, one of them being complicated with cerebral affection. Ague, which was very prevalent in the spring quarter, seems almost to have disappeared for a time. Eight cases were reported in the month of June, the greater number of them being of the tertian type. Only one case was noticed during the last two months. Of typhus only one case was returned, and that proved fatal. Of erysipelas, about the average number of cases appeared. It was generally of a mild character. The furunculoid diseases were rather more common than usual. Some very severe carbuncles were noticed."

*Abstract of Meteorological Observations for the Summer Quarter*, 1857.

| | *June.* Deg. | *July.* Deg. | *August.* Deg. |
|---|---|---|---|
| Highest temperature in the day time .... | 75 | 76 | 76 |
| Lowest .............................. | 57 | 58 | 51 |
| Mean ................................ | 64·96 | 66·32 | 63·12 |
| Highest temperature at night .......... | 63 | — | 65 |
| Lowest .............................. | 40 | — | 50 |
| Mean ................................ | 52·5 | — | 56·41 |
| Highest reading of barometer .......... | 30·26 | 30·23 | 30·20 |
| Lowest .............................. | 29·55 | 29·61 | 29·64 |
| Mean ................................ | 29·925 | 29·884 | 29·910 |
| Number of days on which rain fell ...... | 9 | 9 | 12 |
| Amount of rain in inches .............. | 1·16 | 1·13 | 2·23 |

Direction of wind (the number indicates the number of days certain winds prevailed). *June*—N. 6, NE. 3, E. 8, S. 3, SW. 6, W. 4. *July*—N. 1, NE. 3, S. 1, SW. 13, W. 9, NW. 4. *August*—N. 5, NE. 3, E. 5, SE. 2, S. 2, SW. 7, W. 5, NW. 2.

*Chatham.*—Dr. Brown says: "The diarrhœa cases were abundant during the period embraced in this report. The cholera cases were not severe. Typhoid fever continued to be prevalent, but the mortality diminished in its ratio to the number of seizures. The fever continued to be marked by cephalic complications and by hæmorrhages, epistaxis, and intestinal bleeding. Sequelæ were common, and ague attacked many of the convalescents. There was a marked absence of scarlatina and measles, and no epidemic of influenza. Ague, in all its forms, continued to prevail. Two cases ended fatally in the last week of August, one by coma, the other by quasi-delirium tremens."

*Wandsworth.*—Mr. Nicholas writes: "Diarrhœa here, as elsewhere, was the prevailing epidemic of the quarter. General disease was comparatively very trifling, and, as is observed during the prevalence of epidemic diarrhœa, shewed a great tendency to become complicated with or to emerge into that disease. Since the week ending August 22nd, which was coincident with a lowered temperature, diarrhœa has undergone a gradual diminution both in extent and severity. Although it has been extensive and of considerable severity, it has been attended with little mortality. This, perhaps, may be in a measure due to a greater tractability of the disease, but is more probably attributable to the early application for medical assistance, which is now adopted by the public generally."

*Putney.*—Mr. Whiteman writes: "Diarrhœa was almost the only disease of the zymotic class that prevailed here with any great intensity during the quarter. Some of these cases were of a very aggravated character, and two exhibited all the symptoms of cholera that marked the fatal form of the disease in 1848-9 and 1854. The disease throughout was, however, singularly amenable to treatment, and but very few deaths resulted; the patients who succumbed having been, for the most part, the very young or the very aged. The case of ague that occurred in the week ending July 10th presented some peculiar features. The subject of this attack was a man who, it was ascertained, had not been exposed to any of those influences supposed to cause or to aggravate the disease; but his wife had some time before contracted the disease at Sheerness, where she had been on a visit. She had, however, quite recovered the attack, and had been exceedingly well for several weeks previously to the husband being taken ill. Arsenic was tried in this case without any beneficial result, but quinine in large doses quickly destroyed the periodicity of the disease and restored the patient to health. The diminution of uric acid in the urine was very marked upon the decline of the disease."

*Swansea.*—Mr. Michael says: "Three further cases of smallpox occurred. One was the servant girl in the house where one of the former cases resided; another was a case of confluent small-pox (fatal) from an infected district; and the other was a very slight case, also imported. The health for the first quarter was good; but diarrhœa of a serious character in some cases was very prevalent. It ran to cases of cholera: one died from exhaustion, and one from consecutive fever. Several cases of dysentery also occurred."

*Aspley Guise.*—Dr. J. Williams writes: "The case of ague I have reported continued many weeks, and was of a severe and obstinate character. It occurred in a poor man, who had recently recovered from a scald on the foot. The exciting cause appeared to be great exertion in *threshing* in an open barn in windy, changeable weather, and that also in a weak state of the system from recent illness. The locality was Salford—a damp, undrained village, though situated on table land. The water is bad, and the mortality high—the average for seven years, ending 1853, being thirty per thousand. He had severe pain in the back, and frequent delirium from congestion of the brain following the cold stage. Ipecacuanha emetics, with calomel purges, and salines, with tartar emetic, were of the most service. Warm baths assisted materially in shortening the cold stage. Stimulants were injurious, as also quina and cinchona, arsenic, etc., even when debility had considerably advanced. The urine was highly charged with uric acid greater part of the time, and occasionally contained a large quantity of bile. I must not omit to mention that the urine was entirely suppressed on several occasions, and at that time the pain in the back was distressing, no doubt from congestion of the kidneys. When reaction was established, the perspiration, like all the other symptoms, was excessive, and saturated the linen and bed-clothes through. He ultimately did well, but the case extended over two months.

"During the month of June I have had to treat eight or ten cases of stomatitis or simple *muguet.* In all instances the disease has occurred in large families with *over-crowded bed-rooms,* though, as remarked by MM. Baron and Billard, it did not seem contagious. The subjects were feeble lymphatic children. There was considerable fever attending the disease; which, in some of the cases, continued for three weeks or more, in most of them the breath was extremely offensive, and a brown membranous exudation covered the teeth and gums, similar to the sordes of typhoid fever. Ipecacuhana emetics, and antimonial salines, were of most service, and calomel or grey powder in alterative doses. A lotion of biborate of soda with honey appeared useful; and in the worst cases attended with fœtor, a weak solution of chloride of zinc seemed of the greatest service. How much is it to be regretted that the admission of fresh air by open windows during the night is not more enforced! To the close, foul bedrooms of the poor may be traced at least *half the illness they suffer;* and it should not be forgotten, that by the open window *at all times,* we double and treble the area of *breathing space* for the inmates, and sweep away the active agent, foul air, which is hourly saturating their blood and predisposing them to disease."

*Saffron Walden.*—Mr. Stear says that the quarter was a very healthy one; but diarrhœa was very prevalent, especially during the month of August. He had not a fatal case. The summer has been very hot and dry; to the latter cause he is inclined to attribute the little illness there has been. The harvest has been above the average.

*Wellingborough.*—Mr. Dulley says that all the cases of "typhus" assumed rather the typhoid character, and occurred in those neighbourhoods where the drainage was bad.

*Thetford.*—Mr. Bailey sends the following observations for the quarter: "June. This was an unusually hot month, the temperature being above summer heat during half the month, reaching to 89° on the 28th, and during midnight not lower than temperate; the barometer fluctuating between 26·0° and 30·3°, accompanied by little rain; yet we had comparatively but few diseases under treatment. Four cases of modified small-pox occurred in a neighbouring village; the disease was brought by a servant leaving London, and thus communicated to her immediate neighbours. One man and some children in a closely inhabited spot took the disease, but in so modified a state as scarcely to require medical attendance; these had been previously vaccinated, and two infants were immediately vaccinated and escaped entirely from the disease. It spread no further from this spot. Catarrhs and some cases of influenza became rather prevalent from the second week, which probably arose from the sudden reduction of temperature, together with a north-east wind; there being a difference of temperature of 20°. One delicate little boy was suddenly attacked with croup, and, although the most active measures were resorted to, he sank in two days. Ague and remittent fever were more prevalent than usual this month. Only one case of diarrhœa occurred, which came under notice the last day of the month, and seemed to arise from the excessive heat on the 28th. In the case of typhus which occurred in May, a relapse took place; and after a few days it terminated fatally in the union workhouse. The casual diseases under notice were hæmoptysis, menorrhagia, atrophy, tic douloureux, hysteria, dyspepsia, hemicrania, and petechiæ. The prevalent diseases amongst cattle were pleuropneumonia, sore throat, and some cases of enteritis. Vegetation was very luxuriant and prematurely forward.

"July. The temperature of this month continued exceedingly high from the 11th to the end. During the first week it did not reach higher than 70°, excepting on the 5th, when it rose to 76° ; its lowest point being 54°, a difference of 24° in five hours. The quantity of rain during the month barely exceeded half an inch. The highest temperature was on the 15th at 88°, and lowest on the 5th at 54°. The barometer fluctuated but little ; the mean being 29·52°. The mean of midnight temperature was 63°, and the mean of dew-point 59°. The diseases of this month were but few. Only two cases of small-pox occurred, which required no treatment. Probably but for the close confinement of the rooms and the huddling together of the children, the disease would not have extended beyond the first case. Croup occurred in two cases ; one proved fatal a few hours after I was called to it ; the other recovered. For a number of years I have never witnessed so many cases as within the last twelve months ; it might be considered epidemic. Several cases occurred under the neighbouring medical men. Diarrhœa was more prevalent, attacking indiscriminately all grades and producing great prostration. Fever of a low type occurred amongst labouring men ; one case terminating fatally and the other slowly recovering from the great debility. The case of carbuncle was in an elderly man, at the back of the neck, of a large size ; being early incised, it soon suppurated, and did well in a short time. Rheumatism, both acute and chronic, was more prevalent this month : and many cases of cynanche tonsillaris arose from imprudent exposure. Chicken-pox was quite epidemic this month, going through many families in various villages and in this town. The casual diseases under treatment were amenorrhœa, malignant ulcer of the os uteri, anæmia, leucorrhœa, iritis, syphilitic eruption, pulmonary congestion, spina bifida, lepra vulgaris, porrigo, and erythema. But few diseases, and those only of a casual nature, came under treatment among cattle. Fruits and vegetables became prematurely ripe ; and potatoes underwent a second growth.

"August. This month commenced with a high temperature; being 10° higher than summer heat ; but on the 5th and 6th days very heavy rains fell, amounting to more than fell during the previous three months. This reduced the temperature

12°, which transition was much felt. The barometrical range fluctuated but little during the month; there were only eight windy days. Small-pox and chicken-pox had entirely left the neighbourhood. From the excessive heat of the weather, diarrhœa was extraordinarily prevalent, attacking all grades of society, and in some instances assuming the cholera type. One poor labouring man, who had been affected with diarrhœa some days, although he kept to his work, was suddenly seized with cramp, marble coldness of the whole surface of the body, and violent abdominal pains; the tongue was cold and covered with a white mucus; the stools were completely colourless and running away from him; the pulse was almost imperceptible and very quick; prostration was extreme, and he was apparently insensible to those around him. Brandy was administered, with opium, calomel, and antimony; and a mixture of chlorate of potash, aromatic confection, chloric æther, and acacia powder, in water, in a mixture. This in a few hours brought on a reaction, and he recovered much. The following day he was much improved, but a relapse took place with an increase of all the symptoms, and he sunk in a few hours. In many cases the disease was very severe, occasioning in a few hours the greatest debility I ever witnessed. Scarcely a family escaped the epidemic; and some young infants fell victims to it. Three cases of typhoid fever in one family are under medical treatment: they are labourers, and during the heat they drank largely of small beer and exposed themselves to the currents of air. These cases commenced with diarrhœa, after which fever came on; and they are now under treatment. A case of erysipelas occurred in a woman, attacking the head and face, with smart fever; it was supposed to be brought on by the heat of the weather. Under the usual mode of treatment the case recovered quickly. The casual diseases were neurosis, cardialgia, gout, hysteria, spinal disease, and cancer uteri."

*Record of Deaths in Twenty-one Parishes (population* 9,574) *from the Registrar's Book.*

| *June.* | | *July.* | | *August.* | |
|---|---|---|---|---|---|
| Scarlatina | 1 | Consumption | 1 | Diarrhœa | 3 |
| Fever | 1 | Bronchitis | 2 | Choleraic diarrhœa | 1 |
| Consumption | 3 | Croup | 2 | Consumption | 1 |
| Convulsions | 1 | Diseased heart | 1 | Asthma | 1 |
| Congenital obstruction | 1 | Convulsions | 4 | Debility | 5 |
| Laceration of bowel | 1 | Spasms | 1 | | |
| Dropsy | 3 | Enteritis | 1 | | |
| Atrophy | 1 | Dis. prostate gland | 1 | | |
| Debility | 2 | Atrophy | 1 | | |
| Decay of nature | 1 | Debility | 2 | | |
| Drowned | 1 | | | | |
| | 16 | | 16 | | 11 |

*Dudley.*—Mr. Houghton says: "There was a very slight decrease in small-pox during the thirteen weeks; and 22 deaths only occurred, against 28 in the last quarter. Measles, too, slightly declined; they caused 12 deaths. Deaths from scarlet fever increased: six persons died: it carried off only two last quarter. Fever, hooping-cough, and croup were nearly the same number as in last quarter. Diarrhœa caused 20 deaths; fever, 9; and infantile convulsions, 14. The total deaths were 303: the average for seven corresponding quarters was 216: the excess of deaths above the average was 87. 106 deaths arose from zymotic diseases, being 1 in 2-287. In London, in the corresponding period, the deaths were 13,998; of these, 4,190 were from zymotic diseases, or 1 in 3·204. The deaths in London in the corresponding thirteen weeks were 13,998: the average of the thirteen corresponding quarters was 14,283, the mortality being 283 below the average. Thus Dudley continues to contrast unfavourably with London."

*Barrowden.*—Mr. Swann writes: "Diarrhœa was the most common disease; in many cases it might more properly be designated English cholera. I had two cases of Asiatic cholera; the first one was a most severe case, but has done well; both recovered. Mumps appeared at North Luffenham."

*East Dereham.*—Dr. Vincent thinks diarrhœa has been more prevalent than he can remember since he has been engaged in practice.

*Pontesbury.*—Mr. Eddowes writes: "A young man, just recovered from small-pox, a native of this neighbourhood, and residing in Staffordshire, returned home in May last. He came by rail to Shrewsbury, a distance of about twenty-five miles; thence he rode home, about fourteen miles, in a carrier's cart. Neither the owner of the cart, nor any of his five children, had been vaccinated. His wife had been vaccinated about thirty years ago. The wife and one son were with the cart on that day. The son, about fourteen years of age, about nine days afterwards, sickened with small-pox. About a fortnight afterwards, three other children sickened; and a few days afterwards the father sickened also. One son was in service; and, hearing of his father being dangerously ill with small-pox, went to see him; a fortnight afterwards he was ill with the same disease. They all recovered. The wife went about: she was never ill a day. They were all convalescent the second week of July. The disease was confined to that family, although in a populous neighbourhood."

*Oswestry.*—Mr. Cartwright says: "Small-pox was brought from Bilston, and confined itself to the locality to which it was brought. The diarrhœa was choleraic, with cramps and rice water evacuations. Croup in the early part of the quarter was frequent, and in several cases fatal. Cattle disease (pleuro-pneumonia) was fatal in the locality of the small-pox, but principally upon ill drained farms, and in dirty farm buildings."

*Wrexham.*—Dr. E. Williams writes: "The small-pox cases (seven) were all in one house, and I did not hear of it spreading in the neighbourhood, which was rather singular. The patients had all been vaccinated in childhood, and only one of the cases proved severe (confluent), the others being very mild. The infection was traced to a school one of the children attended."

*Lincoln.*—Mr. Lowe says: "The present quarter has been more than usually unhealthy, the month of August especially; owing, no doubt, to the sudden, heavy fall of rain, followed by extreme heat, which we had at the commencement of the month. We had two cases of scarlet fever, both fatal in sixty hours; also several cases of croup, dysentery, and diarrhœa, and much fever of a low continued character, seldom fatal. In many cases of continued fever the tongue and fauces were covered with aphthæ, which, under the microscope, exhibited a true fungoid character; in one family three patients presented the same appearances."

*Gainsborough.*—Dr. Mackinder says: "Scarlatina has been intruding itself among us, but, as yet, has only got admission into a few establishments. I have had a few cases of the simple and anginose forms. Inflammation of the fauces and tonsils, diphtherite, and stomatitis, have occurred; and there have been two fatal cases of malignant quinsy. Of hooping cough I have seen but one case introduced; and one of carbuncle in a poor man. Chest affections were rare in the quarter; of common continued fever there were a few cases only; and of erysipelas I can number but five patients. From diarrhœa and dysentery I have seen a few sufferers, and have been called to three sharp cases of English cholera. Ague is unknown to us endemically; but a few of our patients have suffered from a mild fever, which assumed an intermittent character, and required quinine or arsenic to establish convalescence. Upon the whole we are among the more favoured localities, in a sanitary point of view; and now that our Board of Health has commenced in right good earnest to redrain the town, we anticipate so long a visit from the fair goddess Hygeia, that the sons of Esculapius entertain serious thoughts of emigrating. The decomposition of vegetable matter, resulting from the recent floods around us, may, however, bring a little work, should the inauspicious winds spread the noxious emanations over our town. With the exception of a few hours, since the great fall of rain, the air has been moving from the north and east points, and so wafted the insalubrious agents in a diluted form to more remote districts. Many acres of potatoes have been rendered worse than useless; and though the corn in our immediate neighbourhood has not deteriorated much, scores of acres of grass have been under water. Apples and pears are plentiful, but wall fruit scarce. Among cattle pleuropneumonia has been epidemic for the last five years; and for some time past there has been common a disease of the influenza character, affecting the mucous membranes, and ending, in horses, with a peculiar disease of the feet. These diseases have not been aggravated by the floods."

The subjoined Meteorological Report is furnished by Mr. Dyson:—"The past quarter was marked by an almost uninterrupted continuance of fine weather, the temperature generally being above the average. The highest range was 92° in the last week of June. Thunder storms, accompanied by a heavy fall of rain, occurred in the first week in July and the second week in August. Between the 6th and 13th of August, the fall of rain was unprecedented, being upwards of seven inches, or fully a quarter of a year's supply in eight days. The development of ozone was considerable during the month of August, but partial during the remainder of the quarter. We have had on the whole what may be aptly termed a 'tropical summer,' excessive heat and severe thunder storms. Meanwhile the general health of the town and neighbourhood was good, the number of deaths being remarkably small."

*Liverpool.*—Mr. Bickerton informs us that during the quarter which ended on June 27th, the total deaths registered in the borough were 2,840, being 390 less than the corrected average of the same quarter of the preceding nine years. The mortality from zymotic diseases was likewise below the average, the deaths being 528, while the mean of the former corresponding quarter was 737. As compared with the quarter immediately preceding, there was a decrease of 40. The only diseases of this class showing an increase during the quarter were hooping cough, which rose from 100 to 158; typhus, which rose from 58 to 78; and diarrhœa, which rose from 49 to 61. In the other diseases of this class the mortality was lower than in the preceding three months; scarlatina fell from 119 to 94; small-pox from 46 to 29; syphilis from 32 to 20; measles from 73 to 11 (less than in any previous quarter). Of

### MORTALITY AND METEOROLOGY OF LIVERPOOL.

| DEATHS. Week ending | June. 6th. | June. 13th. | June. 20th. | June. 27th. | July. 4th. | July. 11th. | July. 18th. | July. 25th. | August. 1st. | August. 8th. | August. 15th. | August. 22nd. | August. 29th. |
|---|---|---|---|---|---|---|---|---|---|---|---|---|---|
| Scarlet fever | 5 | 9 | 5 | 8 | 10 | 3 | 6 | 13 | 11 | 6 | 17 | 14 | 24 |
| Measles | 2 | 1 | 2 | 2 | .. | .. | .. | 2 | .. | 2 | 1 | 2 | 1 |
| Small-pox | 4 | .. | 2 | 1 | 2 | 3 | 4 | 1 | 5 | 1 | 5 | 5 | 4 |
| Hooping cough | 13 | 17 | 17 | 13 | 16 | 10 | 15 | 21 | 13 | 15 | 15 | 19 | 15 |
| Croup | .. | 1 | .. | .. | .. | .. | .. | .. | .. | .. | .. | .. | .. |
| Erysipelas | .. | .. | .. | 2 | .. | .. | .. | .. | .. | .. | .. | .. | .. |
| Cholera* | .. | .. | .. | .. | 2 | .. | 3 | 2 | 8 | .. | 6 | 1 | 2 |
| Diarrhœa† | 4 | 5 | 2 | 12 | 19 | 26 | 23 | 45 | 54 | 54 | 55 | 63 | 73 |
| Typhus | 7 | 8 | 6 | 2 | 12 | 6 | 3 | 5 | 8 | 7 | 6 | 8 | 4 |
| Disease of lungs | 51 | 73 | 69 | 49 | 61 | 54 | 64 | 44 | 66 | 36 | 51 | 48 | 43 |
| Purpura | .. | 1 | .. | .. | .. | .. | .. | .. | .. | 2 | .. | .. | .. |
| Intemperance | 1 | 1 | 1 | 3 | .. | 2 | .. | 1 | .. | .. | .. | .. | .. |
| Syphilis | .. | .. | 2 | 4 | .. | 1 | .. | .. | 2 | .. | 1 | .. | 1 |
| Total deaths during each week | 173 | 206 | 176 | 188 | 241 | 199 | 214 | 238 | 265 | 233 | 274 | 277 | 277 |
| Corresponding week, 1856 | 176 | .. | 186 | .. | 161 | 176 | 175 | 179 | 201 | 234 | 226 | 252 | 230 |
| METEOROLOGICAL OBSERVATIONS. | | | | | | | | | | | | | |
| Barometer: Mean | 30·02 | 29·87 | 30·13 | 30·23 | 29·82 | 29·9 | 30·18 | 29·92 | 29·99 | 29·91 | 29·96 | 30·12 | 30·12 |
| Dew-point temperature | 52·2 | 48·2 | 50·0 | 58·0 | 54·2 | 51·9 | 58·6 | 59·0 | 55·2 | 55·5 | 59·4 | 60·1 | 56·5 |
| Thermometer: Highest (mean) | 69·3 | 63·1 | 70·2 | 76·3 | 70·8 | 64·9 | 72·0 | 70·2 | 70·8 | 70·5 | 70·2 | 74·7 | 75·7 |
| Lowest (mean) | 53·2 | 52·1 | 51·9 | 60·7 | 57·5 | 55·5 | 58·5 | 60·5 | 59·7 | 58·6 | 58·6 | 60·8 | 60·3 |
| Mean | 61·3 | 57·6 | 61·1 | 68·5 | 64·2 | 60·2 | 65·3 | 65·4 | 65·3 | 64·6 | 64·4 | 67·8 | 68·0 |
| Mean of 11 years | 58·5 | 58·7 | 59·0 | 61·5 | 61·5 | 61·7 | 63·0 | 62·5 | 63·0 | 63·8 | 63·0 | 61·4 | 60·7 |
| Range | 16·1 | 11·0 | 18·3 | 15·6 | 13·4 | 9·4 | 13·5 | 9·7 | 11·2 | 11·9 | 11·5 | 13·9 | 15·4 |
| Mean range, 11 years | 12·8 | 10·9 | 10·9 | 10·9 | 11·9 | 10·8 | 10·7 | 10·7 | 10·2 | 11·3 | 10·3 | 9.7 | 9·9 |
| Rain fell on days | 3 | 4 | 1 | 1 | 6 | 3 | 3 | 4 | 3 | 5 | 5 | 0 | 0 |
| Rain fell during hours | 8·9 | 21·9 | 1·8 | 0·3 | 18·5 | 9·7 | 6·8 | 18·7 | 1·4 | 17·6 | 16·1 | 0 | 0 |
| Amount of rain in inches | 0·63 | 1·45 | 0·08 | 0·05 | 2·38 | 0·40 | 0·33 | 1·05 | 0·28 | 1·15 | 1·88 | 0 | 0 |
| General direction of wind | S. SE. | W. | E. | var. | var. | NW. | S. NW. | W. | S. W. | N. | var. | NW. | SE. |

* The cases of cholera noted on July 4th and 18th, and August 15th, were English cholera. A death from glanders is reported to have taken place in the week ending July 25th. In the week ending August 8th, eleven persons died in Liverpool above 80 years old.

† A large proportion of the deaths from diarrhœa occurred in children under two years of age.

these diseases hooping cough was the only one whose absolute mortality was above the average. Diseases of the lungs caused 897 deaths, the average of the same quarter of former years being 870. The mean temperature of the quarter was nearly $54\frac{1}{4}°$, about one degree higher than the average. Rain fell on 42 days, the total amount being 6·96 inches.

Mr. Bickerton has also furnished a statement of the deaths occurring in each week of June, July, and August; and has forwarded copies of the weekly meteorological results deduced from observations taken at the Liverpool Observatory. Of these elaborate documents, however, we are only able to give a very condensed abstract. (See preceding page.)

*Wigan.*—Mr. Hussey writes: "This quarter there has been very little sickness on the whole, and what is remarkable, with high provisions, my pauper district, including workhouse, was comparatively free from sickness. The maximum heat was 112°. Winds were very variable; but the east wind of late evidently caused more work for practitioners. Potatoes called 'Kemps' are going very fast indeed, and in my family I find there is no economy in purchasing any such, as very nearly one-half are unfit for consumption."

*Bolton-le-Moors.*—Mr. Pendlebury writes: "During the past quarter English cholera prevailed rather extensively, and in many cases proved fatal to the aged and debilitated. That of infants was remarkable from the great severity and obstinacy of the vomiting, which in many cases was soon followed by collapse, aphthæ of the mouth, and death. Scarlet fever was also prevalent in its malignant type, but not extensively. A few cases were observed to be combined in symptoms with those of English cholera, the eruption alone being absent, so as to decide the diagnosis correctly. Cases of small-pox were over the average number. Diarrhœa and dysentery almost universally prevailed. During the month of July it was remarked by a few practitioners that parturient women were unusually prone to take cold or suffer (about the tenth or twelfth day) from more or less acute attacks of peritoneal inflammation. Ordinary fever cases were both few and mild."

*Bramley.*—Mr. Radcliffe says: "Diarrhœa was prevalent among infants and young children during the weeks ending June 19th and 26th, and July 24th. Dysentery was prevalent among young children during the weeks ending June 12th and 26th, July 17th and Aug. 14th. Diarrhœa was prevalent among young children and adults during the weeks ending Aug. 21st and 28th. Within the last fortnight diarrhœa has supervened during the progress of several acute cases of disease. The Rev. J. Rawson, the Registrar of Births and Deaths for this district, has kindly furnished me with the following return of deaths which have occurred from zymotic diseases in Bramley during the quarter. Dysentery 1 (a child, aged 17 months, June); diarrhœa 8. (The whole of the deaths from diarrhœa occurred in the month of August, and among children under 23 months of age); scarlet fever 1 (July); typhoid fever 1 (June); continued fever 2 (August.) Two deaths from scarlet fever occurred in the neighbouring village of Horsforth in June. Scattered cases of epizootic pleuro-pneumonia have occurred among horned cattle, in this and neighbouring districts, in each month of the quarter."

*York.*—Mr. Procter writes: "In the cases of scarlet fever the eruption generally showed itself kindly, and the cases were usually mild. Although in a large number there was considerable affection of the throat, they have done well, and were not (as well as the cases of measles) attended with much fatality. Small-pox was of frequent occurrence in adults who had been vaccinated, whilst children who had more recently undergone that operation in the same family escaped. Is not this to be looked upon as an illustration of the limited protective influence of the cow-pox? Diarrhœa was very prevalent: it was both choleraic and dysenteric in its character, and obstinate. Many cases were followed by low typhoid fever, occasionally fatal. The cases were general, and independent of locality. I have not met with pure dysentery frequently. Skin affections were rather prevalent. The crops are full and healthy and will yield well, though the late heavy rains have caused a very large part to be sprouted. Potatoes will here be universally very bad, from the ravages of the 'epidemic'. On the banks of the Ouse, where they are extensively grown for the London market, large quantities have become diseased during a single night, which were apparently healthy when taken up."

*West Auckland.*—Mr. Todd writes: "During the last quarter the weather was unusually warm and dry, with little wind. During the last two months scarlet fever prevailed here and in the neighbourhood very generally. The epidemic was characterised by acute inflammatory fever, with, in many cases, very severe inflammation of the throat, and, in some cases, inflammation of the brain. Many deaths occurred. Erysipelas was more general than usual, generally of a very severe phlegmonous character, with extensive suppuration of the cellular membrane. One case of this kind occurred in a man aged 55—the arm and hand being affected. His wife nursed him and dressed his arm; she had the disease in a milder form. The inflammation commenced on the back of the right hand in both; the wife being attacked a fortnight after the husband. This looks very like contagion. Diarrhœa was very prevalent, in many cases exhibiting the earlier, and some few the advanced stages of cholera. Being of opinion that this premonitory diarrhœa is the primary stage of cholera, I have paid particular attention to early treatment, and to this I attribute my success, no deaths having taken place."

*Lerwick.*—Dr. Spence writes: "The month of June was unusually fine and warm, the prevailing winds were from the south-west. During July the weather was warmer than usual, with the prevailing wind south-west, but it was very changeable, with frequent high winds and much fog and rain. August was warm but very foggy, there being more or less fog on fourteen days. The weather during the whole quarter was particularly favourable to vegetation, and the crops of every kind were much earlier than usual: during July and the beginning of August mushrooms were uncommonly abundant. There were a few slight cases of measles, which I could not trace to any imported source. There were very numerous cases of a rather anomalous eruptive febrile disease with coryza and sore throat, which did not run the usual course of measles, though occurring at the same time with it. In some cases it might be best described as 'something between measles and scarlet fever', followed by desquamation of the cuticle in branny scales, and resembling what I have read of as *Rötheln*, or German measles. In other cases the eruption was vesicular, something like varicella, from which, however, it was distinguished by the coryza, catarrh, and sore throat: this form I imagine to have been the same as the disease popularly known in some parts of Scotland as the 'Nirles'. I have been informed that this morbilloid disease prevailed very extensively throughout all the Shetland Islands during the spring and summer quarters, and it was said to have attacked some who had previously had regular measles. The case of cholera was one of cholera infantum. Diarrhœa was very prevalent in July, and in some cases was attended with vomiting and cramps. There were three cases of dothenenteric fever in June, occurring in men who had returned from the seal-fishery by way of Peterhead. Though paronychia is not generally considered as an epidemic disease, I have included it, from a belief that it does sometimes prevail epidemically. The number of deaths from all causes was 9; the average age was 49; there being one below five years and three over seventy, and the greatest age was eighty-six. And leaving out a case of pneumonia, which was landed from a ship in a dying state, the average age at death would be fifty-three. The causes were encephaloid cancer, 1; chronic diarrhœa, 1; infantile cholera, 1; phthisis, 1; debility from old age, 1; pneumonia, 1; apoplexy, 2; valvular disease of the heart, 1."

*Rothbury.*—Mr. Summers states that the past quarter was remarkably healthy in this district. With the exception of diarrhœa, no disease prevailed epidemically.

